# Reversible Electrochemical Control over Photoexcited Luminescence of Core/Shell CdSe/ZnS Quantum Dot Film

**DOI:** 10.1186/s11671-017-2398-9

**Published:** 2017-12-16

**Authors:** Bo Li, Meilin Lu, Weilong Liu, Xiaojun Zhu, Xing He, Yanqiang Yang, Qingxin Yang

**Affiliations:** Department of Physics, Harbin Institute of Technology, Harbin, 150001 China

**Keywords:** Core/shell CdSe/ZnS quantum dot, Photoluminescence, Electrochemical control, Core emission, Surface emission

## Abstract

Semiconductor quantum dots (QDs) are widely used in light-emitting diodes and solar cells. Electrochemical modulation is a good way to understand the electrical and optical properties of QDs. In this work, the effects of electrochemical control on photoluminescence (PL) spectra in core/shell CdSe/ZnS QD films are studied. The results show different spectral responses for surface emission and core emission when a negative electrochemical potential is applied: the core emission is redshifted while the surface emission is blueshifted. The former is attributed to the electrostatic expansion of the excitonic wave function, due to the asymmetric distribution of adsorbed cations on the surface of the dots. The latter is attributed to the occupation of lower surface states by the injected electrons, i.e., the photoexcited electrons are more likely to be trapped onto higher surface states, leading to a blueshift of the surface emission. Both the spectral shift and the accompanying PL-quenching processes are reversible by resetting the potential.

## Background

Colloidal semiconductor quantum dots (QDs) have received considerable attention for their applications in the field of optoelectronics [1, 2], light emission [3, 4], and high quantum-efficiency photovoltaic devices [5, 6]. QDs have several important properties, especially their size-tunable optical properties, which originate from quantum confinement and result in significant changes in the effective band gap with relatively small changes in size [7].

However, there are many restrictions to the application of QDs in these technologies due to some photoluminescence (PL)-quenching channels. An important aspect of QDs is the exciton surface trap states, which are inherent to QDs because of the high surface-to-volume ratio [8–10]. The trapping of a hot electron/hole effectively reduces the PL efficiency without shifting the emission spectrum or reducing the lifetime, whereas the trapping of a band-edge exciton reduces the PL lifetime [10]. Another important aspect of QDs is the presence of excitons with an additional electron (negative trion) or hole (positive trion), which leads to PL quenching due to fast nonradiative Auger recombination [10–13]. Trion emission also exhibits a shifted spectrum, short lifetime, and random blinking [14–16].

Investigation of the aforementioned PL quenching and shifting, especially the reversible control of these processes, is of significant interest for both fundamental research and technical applications, which are implemented either by electrochemical electron injection or by ionic adsorption. The pioneering work on the electrochemical control of QDs was performed by Wang et al. [17], leading to the discovery of a mid-infrared absorption corresponding to an intraband transition, a bleach of the visible intraband exciton transition, and quenching of the narrow band-edge PL. Several succeeding studies revealed various interesting optical properties of charged QDs, including the reversible control over the “on” and “off” states [18, 19], the amount of charge injected into a single QD, and the corresponding degree of bleach during absorption [19–22]. Recent investigations suggest that the density of trap states can be determined by the electrochemical control of the Fermi level [23, 24], and the energy level offsets in QD heterojunctions can be accurately determined in situ [25].

In addition to the electrochemical control of charge injection, ionic adsorption is another way to regulate PL properties. It has been reported that an irreversible blueshift in the absorption spectrum results from size and/or structural changes in the QDs due to exothermic adsorption [26], and a reversible blueshift results from the anionic adsorption-enhanced quantum confinement [27]. In the latter case, the adsorbed anions compress the electron wave function in the dots, which makes the dots electronically smaller and causes the corresponding blueshift. However, to the best of our knowledge, the spectral shift caused by cationic adsorption-induced quantum confinement and the experimental evidence of the spectral shift caused by electron injection onto the surface trap states have not been reported thus far.

Herein, we show the redshifted and quenched core emission due to cation adsorption and the blueshifted surface emission due to electron injection onto the surface traps. Both the processes are reversible and are controlled using electrochemical methods.

## Methods/Experimental

Core/shell CdSe/ZnS QDs stabilized with octadecylamine ligands were purchased from Sigma-Aldrich (the center of the PL spectrum is 600 nm). QD films with thickness of about 300 nm were grown on cleaned ITO (indium tin oxide) substrates using the dip-coating method, immersed in a 10 mM 1,7-diaminoheptane anhydrous methanol solution for about 20 s, and baked at 70 °C for half an hour for cross-linking. The homemade three-electrode electrochemical cell consists of a Pt disk as the counter electrode, an Ag wire as the quasi-reference electrode, the ITO substrate as the working electrode, and 0.1 mol/L tetrabutylammonium perchlorate (TBAP) in dimethylformamide (DMF) as the electrolyte solution. The Ag quasi-reference was calibrated with ferrocene/ferrocenium with 0.1 M TBAP in DMF, with the offset of about 65 mV versus a standard hydrogen electrode (SHE). The Fermi level of the QD film is controlled by the electrochemical potential between the ITO and the Ag quasi-reference electrode. The steady-state and time-resolved PL spectra are investigated simultaneously using a fiber-optic spectrometer (Ocean Optics 4400) and a TCSPC (time-correlated single photon counting, resolving capability of 180 ps, PMC-100-1, Becker & Hickl GmbH) system, respectively. The sample is excited by picosecond laser pulses (wavelength 375 nm, pulse duration 60 ps, repetition rate 20 MHz).

## Results and Discussion

### Cyclic Voltammograms and Absorption Spectra

**Fig. 1:**
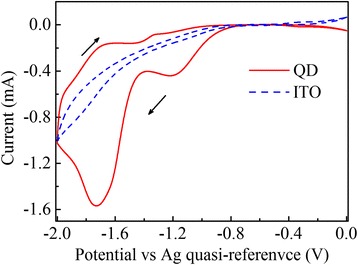
Cyclic voltammetry of CdSe/ZnS QD film (solid) and ITO (dashed) at 100 mV/s. The peak at − 1.7 V is attributed to the charging of 1S_e_ state, and the peak at − 1.2 V is attributed to the charging of surface trap states. The arrows indicate the scan direction. The Ag quasi-reference is offset by 65 mV versus standard hydrogen electrode (SHE)

**Fig. 2:**
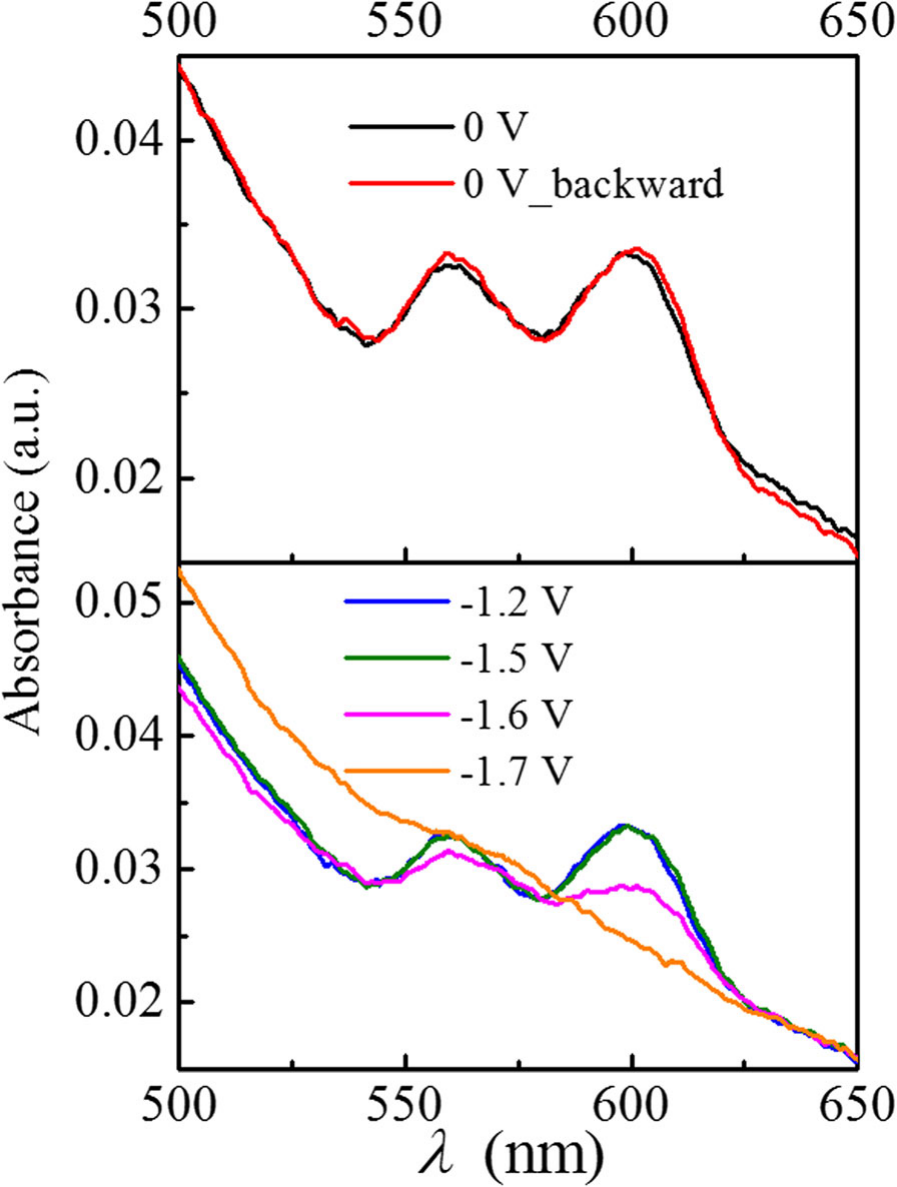
Absorption spectra under representative applied potentials. 0 V (black), − 1.2 V (blue), − 1.5 V (green), − 1.6 V (pink), − 1.7 V (orange), and 0 V backward (red). Two peaks are assigned to the populations of 1S_3/2_1S_e_ and 2S_3/2_1S_e_. Absorbance bleach appears when the applied potential exceeds − 1.6 V and recovers after the potential is reset to zero. Note that the charged QD film (at − 1.6 V) shows a redshift with respect to the uncharged film (at 0 V), and the bleach recovers immediately when the potential is reset to 0 V

Figure 1 shows the cyclic voltammetry of the CdSe/ZnS QD film (solid) and the bare ITO (dashed) at 100 mV/s. The reduction peak (QD film) of − 1.7 V in the scan direction is attributed to the electron injection into the ground excitonic state of the QDs [19]. The electron population of the 1S_e_ state results in a bleach of absorption [17, 18, 22, 28, 29], which is demonstrated in the absorption spectra shown in Fig. 2. The two absorbance peaks of 600 and 560 nm correspond to the transitions of 1S_3/2_1S_e_ and 2S_3/2_1S_e_ [30], respectively, which are intensely bleached at an applied potential of − 1.6 V and completely bleached at − 1.7 V, indicating electron injection into the 1S_e_ state [17, 29]. The partial bleach at − 1.6 V indicates one-electron occupation of the 1S_e_ state [29]. The optical band gap of the QDs at − 1.6 V shows a redshift in the absorption spectrum with respect to that at 0 V, due to the Stark effect in the charged QDs. The bleach recovers immediately as the potential is reset to zero.

In Fig. 1, it can also be seen that the reduction occurs at about − 0.9 V and a reduction peak emerges at − 1.2 V; however, there is no absorption bleach at these potentials (see Fig. 2), which suggests that the electrons are injected into surface trap states rather than excitonic states.

### Time-Resolved/Steady-State PL Under Electrochemical Control

**Fig. 3:**
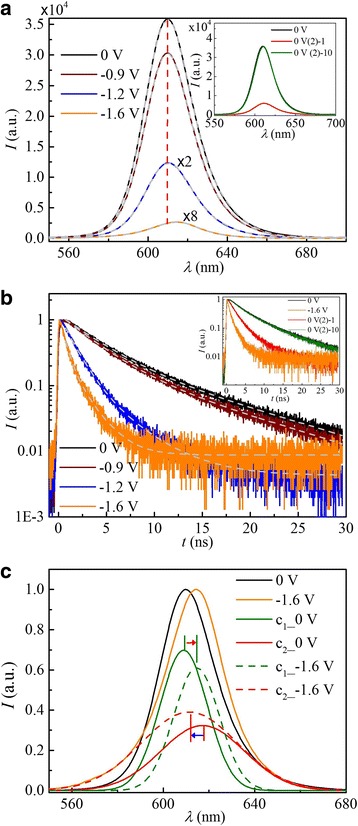
Representative spectra at typical applied potentials. **a** Steady-state spectra and **b** PL decay dynamics. The QDs show a higher PL quantum yield and slower decay rate (black solid) at 0 V. After the applied potential reaches − 0.9 V (wine solid), the PL is quenched with continuous redshift. When the potential is set to − 1.6 V (orange solid), the PL shows considerable quenching. When the potential is reset to 0 V, the spectral intensity and shift exhibit partial recovery instantaneously (inset, 0 V (2)-1, Red solid) and recover completely in ~ 1800 s (inset, 0 V (2)-10, green solid). **c** Normalized PL spectra at 0 V (black solid) and − 1.6 V (orange solid). Each of the two PL spectra can be fit to a sum of two Gaussian functions; the individual Gaussian functions are labeled by c_1_ and c_2_, the solid curves represent the two functions at 0 V, and the dashes represent the two functions at −1.6 V

The steady-state PL spectra and time-resolved PL traces, which are shown in Fig. 3a, b, were simultaneously measured under electrochemical potentials. The spectra captured at potentials of 0, − 0.9, − 1.2, and − 1.6 V are illustrated. As the electrochemical potential is varied from − 0.9 to −1.6 V, the PL emission undergoes remarkable quenching with a progressive redshift and recovers completely in ~ 1800 s at 0 V. It should be noted that the PL emission undergoes an obvious change in line shape with decreasing potential, as shown in Fig. 3c, which will be discussed vide post.

### Excitonic Emission and the Quenching Processes

All the steady-state PL spectra can be fitted to a double Gaussian function (Fig. 3a, gray dash). The high fidelity of the fit suggests that two distinct emitting states are involved. For example, with respect to the QDs at 0 V, each emission component is attributed to the core emission (*λ*_1_ = 609 nm, *w* = 14 nm) and surface emission (*λ*_2_ = 617 nm, *w* = 27 nm). The identification of the core emission and surface emission agrees with the standard model. The surface emission presents a broad spectral width and a redshift with respect to the core emission, due to the broad distribution of the surface-localized trap states and energetically lower levels [31–33].

In order to better understand the spectral redshift and change in line shape, we compare the fitting results of the PL spectra at 0 and − 1.6 V, as shown in Fig. 3c. Here, we assign c_1_ and c_2_ to the core emission and surface emission, respectively. The data clearly indicates that as the potential is reduced to − 1.6 V, the core emission redshifts while the surface emission blueshifts, as indicated by the arrows in the figure.

The two decay components can be well defined by the bi-exponential fitting of the time-resolved spectra (Fig. 3b, gray dashed curves). For example, for the QDs at 0 V, the two components of the PL lifetime are 4.2 and 15.2 ns, respectively assigned to the core emission and surface emission [32–34]. The latter is due to charge transfer to the trap sites located on the shell or at the core/shell interface [35, 36].

**Fig. 4:**
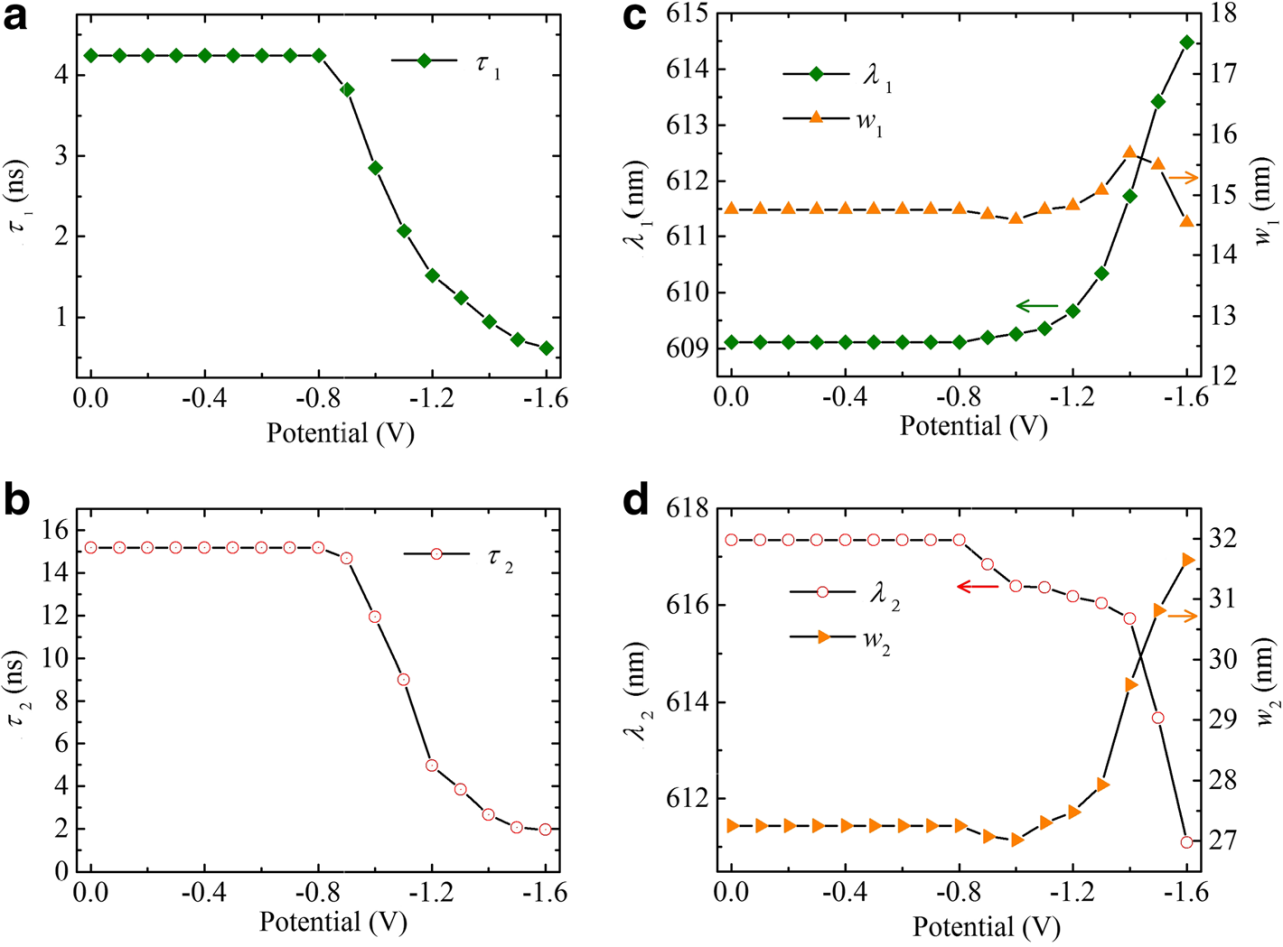
Electrochemical potential dependent fitting parameters of core emission (**a**, **c**) and surface emission (**b**, **d**). The decay times of both the emitting states decrease at an applied potential of − 0.9 V. The core emission shows a redshift (**c**, green squares), and the surface emission shows a blueshift (**d**, red circles). Orange triangles represent the FWHM of the two emission components

The fitting parameters of the time-resolved and steady-state PL spectra for all the applied potentials were plotted as shown in Fig. 4. The core emission lifetime and peak position/spectral width as a function of the applied potential are presented in Fig. 4a and c, respectively. As the applied potential reaches − 0.9 V, the core emission shows a faster decay and a redshifted spectrum relative to that at 0 V. This is attributed to the adsorption of the cations from the electrolyte onto the surfaces of the QDs. As mentioned above, the electron injection into the surface trap states occurs at − 0.9 V. The adsorbed cations serve as counterions to the injected electrons [25, 37, 38] and charge acceptors [39], giving rise to the dissociation of the exciton by charge transfer and quenching of the PL. As the applied potential is decreased to more negative values, a greater quenching occurs due to the potential difference-induced penetration of cations. When the potential becomes − 1.6 V, the 1S_e_ state is occupied by one electron; a negative trion occurs with photoexcitation. The PL is almost completely quenched, mainly due to the large number of nonradiative channels, including effective Auger recombination [10, 40] and considerable charge transfer to the adsorbed cations.

**Fig. 5:**
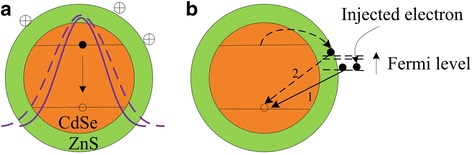
Schematic diagram showing the effects of cation adsorption and electron injection. **a** Effect of cation adsorption on the electronic wavefunction of a photoexcited electron (solid violet curve represents QDs without adsorption and dashed violet curve represents QDs with adsorption). **b** Modulation on surface emission of QDs by injected surface electrons. With increasing negative potential, the lower surface states are progressively occupied by the injected electrons, and the surface emission blueshifts in energy from step 1 to step 2

Previous studies have shown that anions randomly wrapped around QDs cause electrostatic compression of the electronic wave function [27], which makes the dots electronically smaller, resulting in a corresponding blueshift in the PL. In this work, the redshift of the core emission is explained by a reduction in the quantum confinement effect induced by cation adsorption. For the sake of clarity, we plot the model in Fig. 5a. The nonuniform cationic adsorbents on the surface cause electrostatic expansion of the electron wave function in the dot, making the dot electronically larger, leading to a spectral redshift in the core emission.

Note that there are two types of space charge distribution around the QDs: (1) a uniform spherical surface distribution with a radius greater than the exciton Bohr radius, which results in no change to the electronic wave function of the dots, and (2) an inhomogeneous charge distribution around the dots, which can change the excitonic wave function in the dots. In this study, the distribution of cationic adsorbents is considered nonuniform and the uniform charge distribution around the dots, which has not been evidenced so far, is not considered.

The potential dependence of the surface emission lifetime and the peak position/spectral width are presented in Fig. 4b and d, respectively. It can be seen that the surface emission undergoes progressive blueshift and decreasing decay time, which is intimately related to the injected electrons on the surface trap states. This is explained using the model shown in Fig. 5b. With the increase in negative potential beyond − 0.9 V, the Fermi energy increases continuously with the injection of electrons into the lower lying surface states. This traps photoexcited electrons at the higher surface trap states, transforming the surface emission from step 1 to step 2 in Fig. 5b, giving rise to the blueshift. The decreasing decay time of the surface emission may be attributed to the Auger processes present when multiple electrons reside in the surface trap states.

## Conclusions

Electrochemical methods are good tools for understanding the modification of light-emitting properties of QD films by electron injection or ionic adsorption, for both fundamental research and technical applications. Here, we demonstrated the reversible electrochemical control of photoexcited luminescence in core/shell CdSe/ZnS QD films. The results showed that PL quenching occurs after the electrochemical potential reaches a threshold of − 0.9 V (within the band gap), followed by redshifted core emission and blueshifted surface emission, which is reversible in ~ 1800 s after the potential is reset to zero. The redshift of the core emission is attributed to the electrostatic expansion of the electron wave function induced by cation adsorption. On the other hand, the blueshifted surface emission is attributed to the population of surface states. The lower surface states are occupied by injected electrons, and therefore, the photoexcited electrons are more likely to be trapped in the higher surface trap states, leading to the blueshift of the surface emission.

## Abbreviations

DMF: Dimethylformamide
ITO: Indium tin oxide
PL: Photoluminescence
QD: Quantum dot
SHE: Standard hydrogen electrode
TBAP: Tetrabutylammonium perchlorate
TCSPC: Time-correlated single photon counting

## References

[1] Bera D, Qian L, Tseng TK, Holloway PH (2010). Quantum dots and their multimodal applications: a review. Materials.

[2] Eisler HJ, Sundar VC, Bawendi MG, Walsh M, Smith HI, Klimov V (2002). Color-selective semiconductor nanocrystal laser. Appl Phys Lett.

[3] Schlamp MC, Peng X, Alivisatos AP (1997). Improved efficiencies in light emitting diodes made with CdSe (CdS) core/shell type nanocrystals and a semiconducting polymer. J Appl Phys.

[4] Jang I, Kim J, Ippen C, Greco T, Oh MS, Lee J, Kim WK, Wedel A, Han CJ, Park SK (2015). Inverted InP quantum dot light-emitting diodes using low-temperature solution-processed metal–oxide as an electron transport layer. Jpn J Appl Phys.

[5] Nozik AJ (2002). Quantum dot solar cells. Phys E.

[6] Trupke T, Green MA, Würfel J (2002). Improving solar cell efficiencies by down-conversion of high-energy photons. J Appl Phys.

[7] Jones M, Kumar S, Lo SS, Scholes GD (2008). Exciton trapping and recombination in type II CdSe/CdTe nanorod heterostructures. J Phys Chem C.

[8] Kronik L, Ashkenasy N, Leibovitch M, Fefer E, Shapira Y, Gorer S, Hodes G (1998). Surface states and photovoltaic effects in CdSe quantum dot films. Electrochem Soc.

[9] Mao H, Chen J, Wang J, Li Z, Dai N, Zhu Z (2005). Photoluminescence investigation of CdSe quantum dots and the surface state effect. Phys E.

[10] Saba M, Aresti M, Quochi F, Marceddu M, Loi MA, Huang J, Talapin DV, Mura A, Bongiovanni G (2013). Light-induced charged and trap states in colloidal nanocrystals detected by variable pulse rate photoluminescence spectroscopy. ACS Nano.

[11] Galland C, Ghosh Y, Steinbrück A, Sykora M, Hollingsworth JA, Klimov VI, Htoon H (2011). Two types of luminescence blinking revealed by spectroelectrochemistry of single quantum dots. Nature.

[12] Yuan CT, Chou WC, Chuu DS, Chen YN, Lin CA, Chang WH (2008). Photoinduced fluorescence enhancement in colloidal CdSeTe ZnS core/shell quantum dots. Appl Phys Lett.

[13] Efros AL, Rosen M (1997). Random telegraph signal in the photoluminescence intensity of a single quantum dot. Phys Rev Lett.

[14] Shimizu KT, Neuhauser RG, Leatherdale CA, Empedocles SA, Woo WK, Bawendi MG (2001). Blinking statistics in single semiconductor nanocrystal quantum dots. Phys Rev B.

[15] Rosen S, Schwartz O, Oron D (2010). Transient fluorescence of the off state in blinking CdSe/CdS/ZnS semiconductor nanocrystals is not governed by Auger recombination. Phys Rev Lett.

[16] Park YS, Bae WK, Pietryga JM, Klimov VI (2014). Auger recombination of biexcitons and negative and positive trions in individual quantum dots. ACS Nano.

[17] Wang C, Shim M, Guyot-Sionnest P (2001). Electrochromic nanocrystal quantum dots. Science.

[18] Wang C, Shim M, Guyot-Sionnest P (2002). Electrochromic semiconductor nanocrystal films. Appl Phys Lett.

[19] Jha PP, Guyot-Sionnest P (2007). Photoluminescence switching of charged quantum dot films. J Phys Chem C.

[20] Guyot-Sionnest P, Wang C (2003). Fast voltammetric and electrochromic response of semiconductor nanocrystal thin films. J Phys Chem B.

[21] Gooding AK, Gómez DE, Mulvaney P (2008). The effects of electron and hole injection on the photoluminescence of CdSe/CdS/ZnS nanocrystal monolayers. ACS Nano.

[22] Wang C, Wehrenberg BL, Woo CY, Guyot-Sionnest P (2004). Light emission and amplification in charged CdSe quantum dots. J Phys Chem B.

[23] Boehme SC, Walvis TA, Infante I, Grozema FC, Vanmaekelbergh D, Siebbeles LDA, Houtepen AJ (2014). Electrochemical control over photoinduced electron transfer and trapping in CdSe-CdTe quantum-dot solids. ACS Nano.

[24] Boehme SC, Azpiroz JM, Aulin YV, Grozema FC, Vanmaekelbergh D, Siebbeles LDA, Infante I, Houtepen AJ (2015). Density of trap states and Auger-mediated electron trapping in CdTe quantum-dot solids. Nano Lett.

[25] Boehme SC, Vanmaekelbergh D, Evers WH, Siebbeles LDA, Houtepen AJ (2016). In situ spectroelectrochemical determination of energy levels and energy level offsets in quantum-dot heterojunctions. J Phys Chem C.

[26] Landes C, Braun M, Burda C, El-Sayed MA (2001). Observation of large changes in the band gap absorption energy of small CdSe nanoparticles induced by the adsorption of a strong hole acceptor. Nano Lett.

[27] Sarkar SK, Chandrasekharan N, Gorer S, Hodes G (2002). Reversible adsorption-enhanced quantum confinement in semiconductor quantum dots. Appl Phys Lett.

[28] Shim M, Wang C, Guyot-Sionnest P (2001). Charge-tunable optical properties in colloidal semiconductor nanocrystals. J Phys Chem B.

[29] Guyot-Sionnest P (2008). Charging colloidal quantum dots by electrochemistry. Microchim Acta.

[30] Klimov VI, McBranch DW, Leatherdale CA, Bawendi MG (1999). Electron and hole relaxation pathways in semiconductor quantum dots. Phys Rev B.

[31] Mooney J, Krause MM, Saari JI, Kambhampati P (2013). Challenge to the deep-trap model of the surface in semiconductor nanocrystals. Phys Rev B.

[32] Wang X, Qu L, Zhang J, Peng X, Xiao M (2003). Surface-related emission in highly luminescent CdSe quantum dots. Nano Lett.

[33] Morello G, Anni M, Cozzoli PD, Manna L, Cingolani R, Giorgi MD (2007). The role of intrinsic and surface states on the emission properties of colloidal CdSe and CdSe/ZnS quantum dots. Nanoscale Res Lett.

[34] Knowles KE, Mcarthur EA, Weiss EA (2011). A multi-timescale map of radiative and nonradiative decay pathways for excitons in CdSe quantum dots. ACS Nano.

[35] Minotto A, Todescato F, Fortunati I, Signorini R, Jasieniak JJ, Bozio R (2014). Role of core-shell interfaces on exciton recombination in CdSe-Cd_x_Zn_1-x_S quantum dots. J Phys Chem C.

[36] Califano M, Franceschetti A, Zunger A (2005). Temperature dependence of excitonic radiative decay in CdSe quantum dots: the role of surface hole traps. Nano let.

[37] Boettcher SW, Schierhorn M, Strandwitz NC, Lonergan MC, Stucky GD (2010). Ionic-ligand-mediated electrochemical charging of anionic gold nanoparticle films and anionic-cationic gold nanoparticle bilayers. J Phys Chem C.

[38] Boehme SC, Wang H, Siebbeles LDA, Vanmaekelbergh D, Houtepen AJ (2013). Electrochemical charging of CdSe quantum dot films: dependence on void size and counterion proximity. ACS Nano.

[39] Edme K, Homan SB, Nepomnyashchii AB, Weiss EA (2016). Ultrafast exciton decay in PbS quantum dots through simultaneous electron and hole recombination with a surface-localized ion pair. Chem Phys.

[40] Jha PP, Guyot-Sionnest P (2009). Trion decay in colloidal quantum dots. ACS Nano.

